# [^68^Ga]-DOTATOC PET/CT Volumetric Parameters Reflect Metastatic Potential in Pancreatic Neuroendocrine Tumors

**DOI:** 10.3390/cancers17091487

**Published:** 2025-04-28

**Authors:** So Jeong Kim, Jongtae Cha, Hee Seung Lee, Moon Jae Chung, Jeong Youp Park, Seungmin Bang, Seung Woo Park, Si Young Song, Arthur Cho, Jung Hyun Jo

**Affiliations:** 1 Division of Gastroenterology and Hepatology, Department of Internal Medicine, Ewha Womans University College of Medicine, Seoul 03760, Republic of Korea; fantastifo7@gmail.com; 2 Graduate School, Yonsei University College of Medicine, Seoul 03722, Republic of Korea; 3 Department of Nuclear Medicine, Severance Hospital, Yonsei University College of Medicine, Seoul 03722, Republic of Korea; itcha@yuhs.ac; 4 Division of Gastroenterology, Department of Internal Medicine, Severance Hospital, Yonsei University College of Medicine, Seoul 03722, Republic of Korea; lhs6865@yuhs.ac (H.S.L.); mjchung@yuhs.ac (M.J.C.); sensass@yuhs.ac (J.Y.P.); bang7028@yuhs.ac (S.B.); swoopark@yuhs.ac (S.W.P.); sysong@yuhs.ac (S.Y.S.); 5 Division of Gastroenterology, Department of Internal Medicine, Gangnam Severance Hospital, Yonsei University College of Medicine, Seoul 06273, Republic of Korea

**Keywords:** [^68^Ga]-DOTATOC PET/CT, pancreatic neuroendocrine tumors, diagnosis, WHO grade, metastasis

## Abstract

**Background:** [^68^Ga]-DOTATOC PET/CT is a valuable technique for identifying neuroendocrine tumors overexpressing somatostatin receptors; however, its diagnostic and prognostic utility for WHO low-grade pancreatic neuroendocrine tumors remains unclear. Therefore, we aimed to evaluate [^68^Ga]-DOTATOC uptake in well-differentiated pancreatic neuroendocrine tumors and determine its predictive capability for metastasis. **Methods:** Patients with pathologically diagnosed well-differentiated, non-functional pancreatic neuroendocrine tumors who underwent [^68^Ga]-DOTATOC PET/CT between 2015 and 2021 were included. Medical records and [^68^Ga]-DOTATOC PET/CT indices (maximal and mean standardized uptake values, somatostatin receptor-expressing tumor volume, and total lesion somatostatin receptor expression in pancreatic tumors) were retrospectively reviewed. Correlations between indices were analyzed to determine their collective diagnostic significance. **Results:** Among 93 patients who were pathologically diagnosed with pancreatic neuroendocrine tumors and underwent [^68^Ga]-DOTATOC PET/CT, 48 with well-differentiated, non-functional pancreatic neuroendocrine tumors without accompanying genetic syndromes were included. The pancreatic neuroendocrine tumors were classified as WHO grade 1 (n = 30, 62.5%) and grade 2 (n = 18, 37.5%), with tumors in 25% of the patients exhibiting initial metastases. A higher incidence of metastasis was observed in larger metabolically active tumors (somatostatin receptor-expressing tumor volume, *p* < 0.001; total lesion somatostatin receptor expression, *p* < 0.001). **Conclusions:** Volumetric parameters derived from [^68^Ga]-DOTATOC PET/CT correlates with initial metastasis in well-differentiated pancreatic neuroendocrine tumors.

## Simple Summary

Determining the risk of metastasis in pancreatic neuroendocrine tumors is critical for guiding the most appropriate therapeutic approach. [^68^Ga]-DOTATOC PET/CT is a valuable technique for identifying pancreatic neuroendocrine tumors overexpressing somatostatin receptors and may provide insights into the biological behavior of pancreatic neuroendocrine tumors. We aimed to evaluate [^68^Ga]-DOTATOC uptake in well-differentiated pancreatic neuroendocrine tumors and determined its predictive capability for metastasis. Data from 48 patients with well-differentiated, non-functional pancreatic neuroendocrine tumors without accompanying genetic syndromes were analysed. A higher incidence of metastasis was observed in larger metabolically active tumors. Our findings may help clinicians make more precise treatment decisions, ultimately benefiting patients with pancreatic neuroendocrine tumors.

## 1. Introduction

Neuroendocrine tumors (NETs) are characterized by the overexpression of somatostatin receptors (SSTRs). Among the identified SSTRs, SSTR2, SSTR3, and SSTR5 are considered the most clinically relevant, given that most available SSTR analogs bind to these receptor subtypes [1,2,3]. This characteristic allows the use of radiolabeled somatostatin analogs in functional imaging of NETs [4,5,6]. Positron emission tomography/computed tomography (PET/CT) using [^68^gallium] labeled somatostatin analogues are replacing traditional ^111^indium pentetreotide scintigraphy owing to more favorable imaging characteristics for staging NETs, with the added advantage of evaluating therapeutic responses [7,8,9,10]. These PET somatostatin analogues have different affinities for SSTR and should be taken into account during radiotracer selection ([^68^Ga]-DOTATOC (SSTR 5), [^68^Ga]-DOTA-NOC (SSTR 3 and 5), and [^68^Ga]-DOTA-TATE (SSTR 2)) [11,12]. To determine the prognosis of patients with NETs, current semiquantitative methods used to estimate [^68^Ga]-DOTATOC uptake in primary tumors include maximum standard uptake value (SUVmax), mean standard uptake value (SUVmean), SSTR-expressing tumor volume (SRETV), and total lesion SSTR expression (TLSRE = SRETV × SUVmean), which have been found to effectively reflect NET characteristics and patient prognosis [13,14,15,16,17,18,19].

Pancreatic NET (pNET) has an exceptionally high risk of metastasis, with small lesions exhibiting a metastasis rate of approximately 15% [20,21,22]. Thus, predicting metastasis at diagnosis is crucial to improve patient prognosis. However, no known clinicopathologic or imaging indices can consistently identify the risk of metastasis in pNETs. Consequently, establishing a metastasis prediction system using [^68^Ga]-DOTATOC PET/CT imaging indices may be of clinical value. However, previous studies on pNETs and [^68^Ga]-DOTATOC PET/CT have predominantly focused on classifying tumors into World Health Organization (WHO) grades 1 (G1), 2 (G2), or 3 (G3). Given that discrepancies primarily occur in intermediate-grade cases, there is a need to further subdivide and assess tumor grades in [^68^Ga]-DOTATOC PET/CT imaging [10,13]. Despite these clinical needs, only a few studies have predicted the clinicopathological characteristics of subgroup pNETs using solely [^68^Ga]-DOTATOC PET/CT indices. Therefore, we aimed to determine whether [^68^Ga]-DOTATOC PET/CT indices correlate with the clinicopathologic factors in well-differentiated (WD) non-functional pNETs as classified according to the 2017 WHO classification of neuroendocrine neoplasms (NENs).

## 2. Materials and Methods

### 2.1. Patients and Study Design

The study protocol adhered to the tenets of the Declaration of Helsinki and was approved by the Institutional Review Board of Severance Hospital (IRB number: 4-2022-1331). Owing to the retrospective study design, the IRB waived the requirement for informed patient consent.

Patients with pathologically confirmed WD and non-functional pNET who underwent [^68^Ga]-DOTATOC PET/CT at Yonsei University Severance Hospital between 2015 and 2021 were included. Patients diagnosed with WHO G3, neuroendocrine carcinoma (NEC), and accompanying syndromes, such as multiple endocrine neoplasia type 1 and von Hippel-Lindau syndrome, were excluded.

We conducted a retrospective review of medical records and imaging data, focusing on clinicopathological factors and [^68^Ga]-DOTATOC PET/CT indices. Subsequently, we identified factors that exhibited a significant correlation between clinicopathological factors and imaging indices of [^68^Ga]-DOTATOC PET/CT.

### 2.2. Clinicopathologic Factors

We reviewed data from clinical records, including age and sex, as well as laboratory data, including serum chromogranin A and cancer antigen (CA 19-9) levels, when available. Tumor size was determined through surgical tissue and imaging analysis, including contrast-enhanced CT. Histopathological characteristics, including the 2017 WHO classification of NENs based on mitotic count and Ki-67 index, were confirmed, along with immunohistochemistry markers such as chromogranin A, cluster of differentiation 56 (CD56), and synaptophysin. Imaging studies, including contrast-enhanced CT, magnetic resonance imaging, and PET/CT, were performed to detect the presence of metastases.

### 2.3. PET/CT Imaging Protocol

PET/CT was performed using a PET/CT scanner (Discovery 710; GE Healthcare, Milwaukee, WI, USA) equipped with 128-slice CT. Prior to imaging, patients fasted for at least 8 h, and clinical charts were reviewed, confirming that no patient had received somatostatin treatment before PET/CT. One hour before image acquisition, [^68^Ga]-DOTATOC was administered intravenously at a dose of approximately 5.5 MBq/kg of body weight. After the initial low-dose CT study (60 mA, 120 kVp), a standard PET protocol was used for scanning from the neck to the proximal thighs, with an acquisition time of 3 min per bed per position in the three-dimensional mode. Images were reconstructed using ordered subset expectation maximization (2 iterations, 16 subsets), and a Gaussian filter of 5-mm FWHM was applied.

### 2.4. [^68^Ga]-DOTATOC PET/CT Analysis

Two experienced nuclear medicine physicians reviewed all PET/CT images using MIM version 7.08 (MIM Software Inc., Cleveland, OH, USA). A spherical volume of interest (VOI) encasing the entire primary tumor was drawn, and a relative threshold of 41% of the SUVmax of the primary tumor was used to define the SRETV. This approach followed the recommendations of the EANM guidelines [23], and the resulting VOIs were considered to reasonably approximate the actual tumor volume. TLSRE was calculated as follows: SRETV × SUVmean.

### 2.5. Statistical Analysis

Normality was checked by the Shapiro–Wilk test for the continuous variables. Descriptive statistics were represented as mean ± SD for the normality-passed variables or median (interquartile range) for the other variables. Variables with normal distributions were analyzed using the Student’s *t*-test, while non-normally distributed variables were analyzed using the Mann–Whitney U test. Clinical data from patients’ samples were analyzed using the χ^2^ and Fisher’s exact tests for categorical data. Spearman’s rank correlation was performed to evaluate the relationship between [^68^Ga]-DOTATOC PET/CT indices and clinicopathologic factors. Receiver operating characteristic (ROC) curve analysis was performed to determine the best cut-off value for predicting metastasis. The Youden index (*J*) was used to determine the cut-off point. All statistical analyses were performed using IBM SPSS Statistics for Windows, version 26.0 (IBM Corp., Armonk, NY, USA). *p* < 0.05 was considered statistically significant.

## 3. Results

### 3.1. Patient Demographics

**Figure 1:**
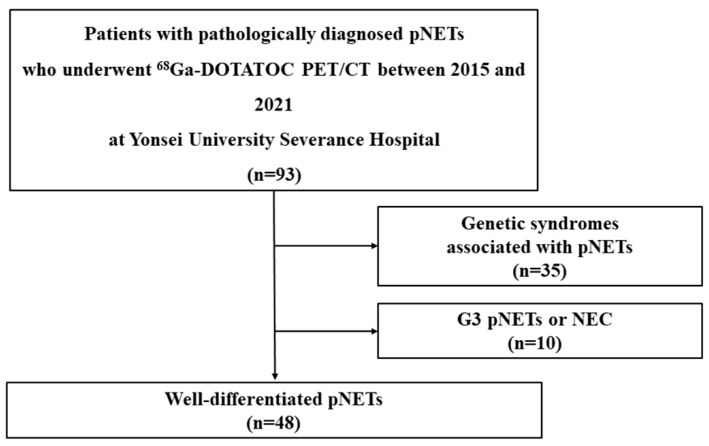
Flow diagram illustrating patient inclusion and exclusion. Of the 93 patients identified, this study involved 48 patients with well-differentiated, non-functional pNETs without genetic syndromes. NEC, neuroendocrine carcinoma; pNET, pancreatic neuroendocrine tumor.

In total, 93 patients with pathologically diagnosed pNETs who underwent [^68^Ga]-DOTATOC PET/CT at our institution between 2015 and 2021 were identified. Among these patients, those with genetic syndromes associated with pNETs (n = 35) and G3 pNETs or NEC (n = 10) were excluded. Finally, 48 patients with WD pNETs were enrolled in our study (Figure 1).

### 3.2. Clinicopathologic Factors and [^68^Ga]-DOTATOC PET/CT Indices According to the Initial Metastasis of pNETs

A total of 48 patients with WD pNETs were enrolled (mean age, 54.81 ± 12.19 years; 27 male patients [56.3%]). Initial metastases were detected in 12 patients (25%) (Table 1). In the non-metastatic group, tumors in most patients (94.4%) were confirmed histologically via surgery, whereas in the metastatic group, tumors in most patients (75.0%) were confirmed via endoscopic ultrasonography fine needle biopsy (*p* < 0.001). Based on laboratory assessments, the metastatic group had higher levels of chromogranin A and CA 19-9 than those in the non-metastatic group, although the difference was non-significant. The metastatic group had significantly larger tumors than the non-metastatic group (34.50 (27.00–40.00) vs. 14.50 (11.00–20.00) mm, *p* < 0.001). Importantly, the metastatic group had a significantly higher proportion of patients with a tumor size of ˃20 mm than the non-metastatic group (91.7% vs. 19.4%, *p* < 0.001). The proportion of G2 tumors and the Ki-67 index, reflecting the tumor grade, were higher in the metastatic group than in the non-metastatic group (75% vs. 25%, *p* = 0.002; 4.90 (2.36–12.00) vs. 1.49 (0.92–2.43), *p* = 0.002). The immunohistochemistry findings, including chromogranin A, CD56, and synaptophysin staining, did not differ significantly between groups (all *p* > 0.05).

**Figure 2:**
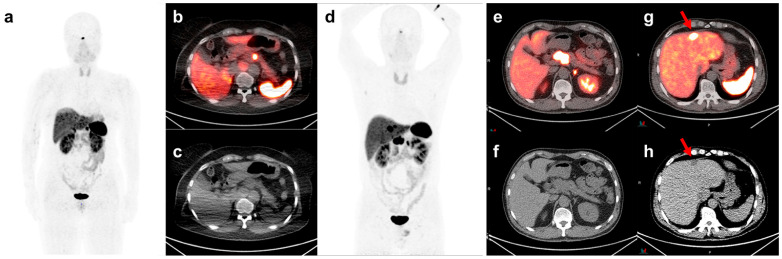
[^68^Ga]-DOTATOC PET/CT maximum intensity projection (MIP), axial fusion, and CT images of: (**a**–**c**) A 60-year-old woman with a G2 pNET located in the pancreatic body and (**d**–**h**) A 51-year-old man diagnosed with G2 pNET. The primary tumor in the pancreatic head is noted (**e**,**f**). Hepatic metastasis was observed on the axial fusion and CT images ((**g**,**h**) red arrows). The metabolic volume of the primary tumor was higher in the patient with initial hepatic metastasis than in the patient without metastasis (TLSRE: 266.96 vs. 16.07, SRETV: 7.23 vs. 0.94). The values of other PET/CT indices were as follows: SUVmax: 65.37 vs. 31.72, SUVmean: 36.91 vs. 17.08. Abbreviations: Ga, Gallium; PET/CT, positron emission tomography/computed tomography; SUV, standardized uptake value; max, maximal; SRETV, somatostatin receptor-expressing tumor volume; TLSRE, total lesion somatostatin receptor expression.

To evaluate the difference in SSTR expression between the metastatic and non-metastatic groups, we compared the mean values of SUVmax and SUVmean, which are linked to the concentration of SSTR-bound [^68^Ga]-DOTATOC, along with SRETV and TLSRE, which are conventionally used to evaluate SSTR-expressing tumor volume. Although no significant differences in SUVmax and SUVmean were detected, the metastasis group exhibited higher SRETV and TLSRE values than the non-metastasis group (11.80 (6.02–16.01) vs. 1.50 (0.84–3.78), *p* < 0.001; 357.14 (290.16–538.25) vs. 41.29 (16.94–67.10), *p* < 0.001, respectively). A representative case is presented in Figure 2.

### 3.3. Diagnostic Performance of [^68^Ga]-DOTATOC PET/CT Indices to Predict Initial Metastasis

**Figure 3:**
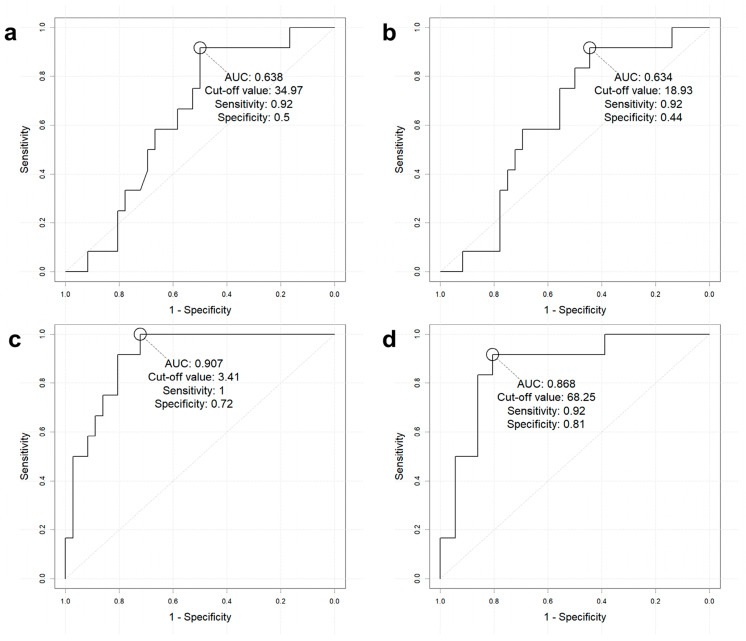
ROC curve analysis for [^68^Ga]-DOTATOC PET/CT indices in predicting initial metastasis. (**a**) SUVmax, (**b**) SUVmean, (**c**) SRETV, and (**d**) TLSRE. Abbreviations: Ga, gallium; PET/CT, positron emission tomography/computed tomography; SUV, standardized uptake value; max, maximal; SRETV, somatostatin receptor-expressing tumor volume; TLSRE, total lesion somatostatin receptor expression.

To evaluate the clinical usefulness of [^68^Ga]-DOTATOC PET/CT indices in predicting initial metastasis, an ROC curve analysis was performed (Figure 3). ROC-generated cut-offs of SUVmax > 34.97, SUVmean > 18.93, SRETV > 3.41, and TLSRE > 68.25 were used to evaluate diagnostic performance in predicting initial metastasis.

Overall, SUVmax and SUVmean showed a similar area under the curve (AUC), high sensitivity (both 91.7% (11/12)), and negative predictive value (NPV) (94.7% (18/19), 94.1% (16/17), respectively; Table 2). Conversely, PET metrics incorporating volume showed a higher AUC than SUVmax and SUVmean (SRETV: AUC 90.7%, TLSRE: 86.8%), along with higher sensitivity (SRETV: 100%, TLSRE: 91.7%, respectively) and higher NPV (SRETV: 100%, TLSRE: 96.7%).

### 3.4. Subgroup Analysis of [^68^Ga]-DOTATOC PET/CT Indices to Predict Metastasis in pNETs Measuring > 20 mm

In our patient population, CT-measured tumor size with a cut-off value of >20 mm showed high sensitivity (91.7%) and high specificity (80.6%) in predicting metastasis (Table 1). However, 7 out of 18 patients (38.9%) with tumors ˃20 mm did not have metastasis, indicating the clinical limitations of using surgical resection based on size criteria alone. Moreover, PET indices showed comparable sensitivity (91.7%) to the CT-measured tumor size in predicting metastasis. Importantly, we found that the accuracy of all PET indices ranged between 72.2% and 77.8% in terms of predicting metastasis of tumors ˃20 mm (Table 2). In tumors ˃20 mm, SRETV presented the highest accuracy (77.8%; 14/18) in predicting metastasis, accompanied by a sensitivity of 100% (11/11) when using the same cut-off of 3.41 cm^3^. Compared to SRETV, TLSRE miscategorized one metastasis (Table 2). Other PET indices revealed similar accuracy but lower sensitivity than SRETV.

### 3.5. Clinicopathologic Factors and [^68^Ga]-DOTATOC Indices According to pNET WHO Grades

Among the 48 patients, the tumors in 30 (62.5%) and 18 (37.5%) were categorized as WHO G1 and G2, respectively (Table 3). The G1 pNET group comprised a higher proportion of male individuals than the G2 pNET group (G1 vs. G2: 70.0% vs. 33.3%, *p* = 0.013). Additionally, a higher proportion of patients in the G1 pNET group underwent surgery for histologic confirmation than that in the G2 pNET group (G1 vs. G2: 90.0% vs. 55.6%, *p* = 0.006). The G2 pNET group had larger tumors (13.00 [11.00–20.00] vs. 23.50 (18.00–35.00), *p* = 0.002) and exhibited metastasis more frequently than the G1 pNET group (10.0% vs. 50.0%, *p* = 0.005). There were no differences between the G1 and G2 pNET groups in laboratory test results or immunohistochemistry findings (serum levels of chromogranin A, CA 19-9, CD56, and synaptophysin, all *p* > 0.05). Upon evaluating [^68^Ga]-DOTATOC PET/CT indices, no significant differences in SUVmax, SUVmean, SRETV, and TLSRE were detected between the G1 and G2 pNET groups.

### 3.6. Relationship Between [^68^Ga]-DOTATOC PET/CT Indices, Proliferative Index, and Serum Markers

Considering that [^68^Ga]-DOTATOC targets somatostatin expression, a higher uptake should reflect more functional tumors. Therefore, to evaluate which [^68^Ga]-DOTATOC indices best reflect tumor function, we performed a Spearman’s rank correlation analysis of known clinicopathological variables that reflect pNET function (Table 4). As summarized in Table 4, none of the [^68^Ga]-DOTATOC-derived indices demonstrated a statistically significant correlation with mitotic count, Ki-67 index, chromogranin A level, or CA 19-9. Although SRETV showed a weak positive correlation with Ki-67 (rho = 0.263, *p* = 0.071), this did not reach statistical significance.

## 4. Discussion

To the best of our knowledge, this is the first study to evaluate the relationship between volumetric [^68^Ga]-DOTATOC PET/CT indices and the presence of initial metastasis in patients with pNETs. Initial metastasis was more accurately predicted by the volume of SSTR-expressing tumors than by the degree of SSTR expression alone. Unlike SUVmax and SUVmean, which reflect only peak or average uptake in localized regions, PET indices such as SRETV and TLSRE reflect both tumor size and function, and has the added advantage of summation of multiple lesions. This allows for a more comprehensive assessment of total tumor burden and is in line with previous reports indicating that only volumetric PET/CT parameters were significantly associated with prognosis [19,24].

We obtained evidence to support the application of non-invasive methods such as [^68^Ga]-DOTATOC PET/CT in patients with WD pNETs to predict initial metastasis. Detecting the presence of metastasis is crucial in determining the treatment strategy for pNETs. A higher rate of metastasis is generally associated with unfavorable pathological characteristics, such as higher grades and larger tumors. However, in the present study, we observed that 25% of patients with WD pNETs had metastasis at diagnosis. Although patients with WHO G2 tumors showed higher rates of metastasis than those with G1, approximately 10% of patients with G1 pNETs also had metastatic lesions, including one patient (8.3%) with a tumor size of ≤20 mm. These findings are concordant with those of previous studies [25,26], where 20–40% of all patients with pNETs were found to present with metastases at diagnosis, including those with WD pNETs < 20 mm [25,26]. Thus, there is a clinical unmet need to predict metastasis in pNETs, especially WD tumors. We demonstrated that the accuracy of SRETV is comparable to that of size criteria in predicting metastasis but with higher sensitivity (100% vs. 91.7%) and lower specificity (72.2% vs. 80.6%). However, more importantly, we found that in tumors ˃20 mm, the accuracy and sensitivity of SRETV were as high as 77.8% and 100%, respectively, in predicting metastasis, potentially suggesting an additional role of [^68^Ga]-DOTATOC PET/CT in predicting metastasis of lesions ˃20 mm. Current guidelines suggest that pNETs > 20 mm should be resected owing to the higher metastatic potential, regardless of the tumor grade [27]; therefore, this approach is valuable in identifying advanced cases because the current consensus for treatment is based solely on size criteria in localized pNETs. Further prospective studies are needed to validate the findings of the present study.

Tirosh et al. reported that the [^68^Ga]-DOTA-avid tumor volume positively correlated with NET biomarker levels [28]. [^68^Ga]-DOTATOC PET/CT may be a valuable technique for estimating the functional tumor burden, given the lack of clinical indices that correlate with elevated serum chromogranin A level in patients with pNETs. However, in our study, no significant correlations were observed. This discrepancy may be attributed to differences in patient population and the limited sample size. Further studies with larger cohorts would be encouraged.

This study has some limitations. First, this was a retrospective study conducted in a single institution; hence, selection bias is inevitable. However, we included a relatively homogenous patient population encompassing those who had pathologically confirmed G1/G2 WD pNETs. In the future, investigations comprising a larger patient cohort need to be undertaken. Second, owing to the retrospective nature of this study, some patients had missing measurement data, such as the proliferative index and serum marker levels, which may have led to limited outcomes. Lastly, although a higher proportion of male patients was observed in the G1 group, this likely reflects a sampling imbalance given the limited number of G2 patients. Previous studies have not demonstrated a consistent association between patient gender and [^68^Ga]-DOTATOC uptake in tumor lesions, suggesting this difference does not confound our imaging-based findings [23].

## 5. Conclusions

Volumetric [^68^Ga]-DOTATOC PET/CT indices, such are SRETV and TLSRE, have added value in predicting initial metastasis in WD pNETs, whereas SUVmax and SUVmean did not. Our findings suggest that patients with larger WD pNETs tumors will benefit from PET/CT in metastasis detection and have an additional role for functional imaging biomarkers in guiding treatment decisions beyond size-based criteria alone.

## Figures and Tables

**Table 1:** Clinicopathologic factors and [^68^Ga]-DOTATOC PET/CT indices according to initial metastasis.

Variable	Total (n = 48)	Non-Metastatic (n = 36)	Metastatic (n = 12)	*p* Value
Age ± SD, years	54.8 ± 12.19	54.3 ± 12.63	56.4 ± 11.12	0.604
Male sex, n (%)	27 (56.3)	21 (58.3)	6 (50.0)	0.614
Histopathologic confirmation method, n (%)				**<0.001**
Operation	37 (77.1)	34 (94.4)	3 (25.0)	
Fine needle biopsy	11 (22.9)	2 (5.6)	9 (75.0)	
Laboratory tests (serum)				
Chromogranin A, median (Q1, Q3), ng/mL	82.75 (38.30, 125.30)	69.70 (19.20, 93.20)	104.15 (63.50, 160.80)	0.079 *
CA 19-9, median (Q1, Q3), U/mL	8.70 (3.70, 13.95)	6.80 (3.70, 13.20)	12.30 (7.90, 16.80)	0.215 *
Characteristics of tumor				
Tumor size, median (Q1, Q3), mm	18.00 (12.00, 27.00)	14.50 (11.00, 20.00)	34.50 (27.00, 40.00)	<0.001 *
Tumor size ≤20:>20, mm, n (%)	30 (62.5):18 (37.5)	29 (80.6):7 (19.4)	1 (8.3):11 (91.7)	**<0.001**
WHO G1:G2, n (%)	30 (62.5):18 (37.5)	27 (75.0):9 (25.0)	3 (25.0):9 (75.0)	**0.002**
Mitotic count, median (Q1, Q3)/50 HPF	1.00 (0.00, 1.00)	0.00 (0.00, 1.00)	1.00 (0.00, 2.00)	0.604 *
Ki-67 index, median (Q1, Q3)	1.98 (1.00, 4.69)	1.49 (0.92, 2.43)	4.90 (2.36, 12.00)	0.002 *
Immunohistochemistry, n (%) †				
Chromogranin A	32 (84.2)	26 (89.7)	6 (66.7)	0.098
CD 56	36 (85.7)	29 (85.3)	7 (87.5)	0.873
Synaptophysin	44 (95.7)	32 (94.1)	12 (100.0)	0.390
[^68^Ga]-DOTATOC PET/CT indices in pancreatic tumor				
SUVmax, median (Q1, Q3)	50.44 (23.37, 78.43)	40.19 (17.96, 70.15)	64.95 (42.72, 85.06)	0.157 *
SUVmean, median (Q1, Q3)	29.14 (13.35, 44.01)	22.69 (9.58, 39.47)	35.48 (24.70, 47.49)	0.167 *
SRETV, median (Q1, Q3), mL	3.25 (0.93, 7.22)	1.50 (0.84, 3.78)	11.80 (6.02, 16.01)	<0.001 *
TLSRE, median (Q1, Q3), g	55.94 (25.82, 318.74)	41.29 (16.94, 67.10)	357.14 (290.16, 538.25)	<0.001 *

* Mann–Whitney U test. † Missing data were excluded from the analysis. Bold = statistically significant (*p* < 0.05). Abbreviations: Ga, gallium; PET/CT, positron emission tomography/computed tomography; WHO, World Health Organization; G, grade; SUV, standardized uptake value; max, maximal; SRETV, somatostatin receptor-expressing tumor volume; TLSRE, total lesion somatostatin receptor expression; SD, standard deviation; Q1, quartile 1; Q3, quartile 3; CA 19-9, cancer antigen 19-9, CD 56, cluster of differentiation 56.

**Table 2:** Diagnostic performance of [^68^Ga]-DOTATOC PET/CT indices in predicting initial metastasis.

	AUC [95% CI]	Group	Sensitivity	Specificity	Accuracy	PPV	NPV
SUVmax >34.97	0.638 (0.486–0.771)	All (n = 48)	91.7% (11/12)	50.0% (18/36)	60.4% (29/48)	37.9% (11/29)	94.7% (18/19)
		Size > 2 cm (n = 18)	90.9% (10/11)	42.9% (3/7)	72.2% (13/18)	71.4% (10/14)	75% (3/4)
SUVmean >18.93	0.634 (0.483–0.768)	All (n = 48)	91.7% (11/12)	44.4% (16/36)	56.3% (27/48)	35.5% (11/31)	94.1% (16/17)
		Size > 2 cm (n = 18)	90.9% (10/11)	42.9% (3/7)	72.2% (13/18)	71.4% (10/14)	75% (3/4)
SRETV >3.41	0.907 (0.788–0.972)	All (n = 48)	100.0% (12/12)	72.2% (26/36)	79.2% (38/48)	54.6% (12/22)	100.0% (26/26)
		Size > 2 cm (n = 18)	100% (11/11)	42.9% (3/7)	77.8% (14/18)	73.3% (11/15)	100% (3/3)
TLSRE >68.25	0.868 (0.739–0.948)	All (n = 48)	91.7% (11/12)	80.6% (29/36)	83.3% (40/48)	61.1% (11/18)	96.7% (29/30)
		Size > 2 cm (n = 18)	90.9% (10/11)	42.9% (3/7)	72.2% (13/18)	71.4% (10/14)	75% (3/4)

Abbreviation—Ga, gallium; PET/CT, positron emission tomography/computed tomography; AUC, area under the curve; CI, confidence interval; PPV, positive predictive value; NPV, negative predictive value; SUV, standardized uptake value; max, maximal; SRETV, somatostatin receptor-expressing tumor volume; TLSRE, total lesion somatostatin receptor expression.

**Table 3:** Clinicopathologic factors and [^68^Ga]-DOTATOC PET/CT indices according to the WHO grades of pNETs.

	WHO Grade 1 (n = 30)	WHO Grade 2 (n = 18)	*p* Value
Age ± SD, year	55.50 ± 12.61	53.67 ± 11.73	0.619
Male sex (%)	21 (70.0)	6 (33.3)	**0.013**
Histopathologic confirmation method, n (%)			**0.006**
Operation	27 (90.0)	10 (55.6)	
Fine needle biopsy	3 (10.0)	8 (44.4)	
Laboratory tests (serum)			
Chromogranin A, median (Q1, Q3), ng/mL	69.70 (35.90, 132.00)	91.15 (42.60, 106.00)	0.907 *
CA 19-9, median (Q1, Q3), U/mL	8.70 (3.70, 14.50)	8.75 (2.85, 13.40)	0.820 *
Characteristics of tumor			
Tumor size, median (Q1, Q3), mm	13.00 (11.00, 20.00)	23.50 (18.00, 35.00)	0.002 *
Tumor size ≤20:>20, mm, n (%)	23 (76.7):7 (23.3)	7 (38.9):11 (61.1)	**0.009**
Metastasis, n (%)	3 (10.0)	9 (50.0)	**0.005**
Mitotic count, median (Q1, Q3)/50 HPF	0.00 (0.00, 0.00)	2.00 (1.00, 3.00)	<0.001 *
Ki-67 index, median (Q1, Q3)	1.00 (0.79, 1.95)	5.70 (4.00, 12.00)	<0.001 *
Immunohistochemistry, n (%) †			
Chromogranin A	23 (92.0)	9 (69.2)	0.154 **
CD 56	24 (85.7)	12 (85.7)	>0.999
Synaptophysin	29 (100.0)	15 (88.2)	0.131 **
[^68^Ga]-DOTATOC PET/CT indices in pancreatic tumor			
SUVmax, median (Q1, Q3)	47.84 (22.76, 68.17)	51.80 (31.72, 84.87)	0.749 *
SUVmean, median (Q1, Q3)	26.23 (12.79, 39.71)	30.17 (17.08, 49.08)	0.733 *
SRETV, median (Q1, Q3), mL	1.83 (0.87, 5.81)	5.31 (1.80, 9.20)	0.092 *
TLSRE, median (Q1, Q3), g	45.35 (24.84, 67.74)	290.16 (35.72, 390.25)	0.136 *

* Mann–Whitney U test. ** Fisher’s exact test. † Missing data were excluded from the analysis. Bold = statistically significant (*p* < 0.05). Abbreviations: Ga, gallium; PET/CT, positron emission tomography/computed tomography; WHO, World Health Organization; G, grade; SUV, standardized uptake value; max, maximal; SRETV, somatostatin receptor-expressing tumor volume; TLSRE, total lesion somatostatin receptor expression; SD, standard deviation; Q1, quartile 1; Q3, quartile 3; pNET, pancreatic neuroendocrine tumor; CA 19-9, cancer antigen 19-9, CD 56, cluster of differentiation 56.

**Table 4:** Relationship among [^68^Ga]-DOTATOC PET/CT indices, proliferative index, and serum markers.

		Mitotic Count * (n = 37)	Ki-67 (n = 48)	Chromogranin A * (n = 24)	CA 19-9 * (n = 36)
SUVmax	rho	0.116	−0.038	−0.116	0.137
	*p*	0.494	0.800	0.589	0.424
SUVmean	rho	0.113	−0.047	−0.144	0.147
	*p*	0.504	0.749	0.503	0.393
SRETV	rho	0.155	0.263	0.133	0.126
	*p*	0.361	0.071	0.537	0.463
TLSRE	rho	0.166	0.199	0.094	0.217
	*p*	0.327	0.175	0.662	0.205

Coefficients of correlation (rho) and *p* values were calculated with two-sided Spearman’s rank correlation test. * Missing data were excluded from the analysis. Abbreviations: Ga, gallium; PET/CT, positron emission tomography/computed tomography; CI, confidence interval; SUV, standardized uptake value; max, maximal; SRETV, somatostatin receptor-expressing tumor volume; TLSRE, total lesion somatostatin receptor expression; CA 19-9, cancer antigen 19-9.

## Data Availability

The data presented in this study are available on request from the corresponding author.

## Abbreviations

[^68^Ga]-DOTATOC, [^68^gallium-DOTA0-Tyr3]octreotide; [^18^F]FDG, [^18^F]fluorodeoxyglucose; AUC, area under the curve; CA 19-9, cancer antigen; CD 56, cluster of differentiation 56; CT, computed tomography; NEC, neuroendocrine carcinoma; NENs, neuroendocrine neoplasms; NPV, negative predictive value; PET, positron-emission tomography; pNETs, pancreatic neuroendocrine tumours; PPV, positive predictive value; SRETV, somatostatin receptor-expressing tumour volume; SSTRs, somatostatin receptors; SUVmax, maximal standardized uptake value; SUVmean, mean standardized uptake value; TLSRE, total lesion somatostatin receptor expression; WD, well-differentiated; WHO, World Health Organization.
